# Malignant Infantile Osteopetrosis: A Case Report

**DOI:** 10.7759/cureus.6725

**Published:** 2020-01-21

**Authors:** Dalal K Bubshait, Ziyad E Himdy, Ola Fadaaq, Hajar I Alshmas

**Affiliations:** 1 Pediatrics, King Fahd University Hospital, Al-Khobar, SAU; 2 Pediatrics, Imam Abdulrahman Bin Faisal University, Dammam, SAU

**Keywords:** osteopetrosis, malignant infantile, autosomal recessive, genetic, hepatosplenomegaly

## Abstract

Osteopetrosis is a rare genetic disease of bone resorption. It includes a variety of hereditary skeletal disorders that have the main radiographic feature of increased bone density and thickness due to differentiation or functional defects in osteoclast. The clinical presentation varies widely based on the type of osteopetrosis and ranges in severity from asymptomatic to a fatal course. Our case is of the infantile malignant osteopetrosis (IMOP) form. It is inherited as an autosomal recessive pattern that generally starts in intrauterine life and manifests at birth or early childhood. It is the most severe form and has an incidence of 1 in 250,000 births. The patient presented at the age of two months with a history of recurrent fever, recurrent pneumonia, developmental delay, and infantile spasms. Upon examination, she was found to have hepatosplenomegaly, axial hypotonia, limb spasticity, and visual impairment. Genetic testing revealed a homozygous variant of OSTM1 gene, which is a known Saudi mutation of autosomal recessive osteopetrosis (ARO). IMOP should be considered as a rare differential of hepatosplenomegaly. Early diagnosis by clinical picture, imaging, and genetic testing is important to direct the appropriate management in order to prevent disease progression before the irreversible neurological sequelae occur. Patients should be managed by a comprehensive approach, and currently, hematopoietic stem cell transplantation (HSCT) provides a better outcome for IMOP patients.

## Introduction

Osteopetrosis is a rare genetic disease of bone resorption. It includes a variety of hereditary skeletal disorders that have the main radiographic feature of increased bone density and thickness due to differentiation or functional defects in osteoclast [1]. Osteopetrosis is known as marble bone disease and Albers-Schönberg was the first who described it in 1904. The two major clinical forms of osteopetrosis are 1) the autosomal dominant adult (benign) type which is considered mild and may be asymptomatic or exhibits few symptoms and, 2) the autosomal recessive infantile (malignant) type. Other forms of osteopetrosis include intermediate recessive type and carbonic anhydrase II deficiency which is associated with renal tubular acidosis and cerebral calcification [2].

Autosomal recessive is the inherited pattern of infantile malignant osteopetrosis (IMOP), most often involves mutations in TCIRG1 (ATP6I) encoding the a3 subunit of the vacuolar proton pump [2]. This type has high severity compared to the above mentioned forms of osteopetrosis and is considered fatal if untreated. Generally it begins in utero and manifests during infancy or early childhood [3]. It is a rare disorder and affects 1 in 250,000 live births, with specifically high incidence recorded in Costa Rica (3.4:100000) [4]. There is a dysregulation and imbalance of bone remodeling as a result of impaired osteoclast function with continued bone thickening and eventually generalized osetoscelerosis [1].

The clinical manifestations are related to the underlying defect in osteoclastic activity and increased bone density. Children exhibit characteristic phenotypic features such as macrocephaly, broad face, and frontal bossing. Tooth eruption is delayed and fractures occur because the bones are dense but fragile, also the child can present with short stature and failure to thrive. Nasal congestion caused by underdevelopment of the mastoid and paranasal sinuses is an early symptom. Neurological manifestations occur due to overgrowth and narrowing of cranial foramina which leads to nerve compression most frequently optic, auditory and facial nerves resulting in blindness, hearing loss, and facial palsy; sometimes patients develop hydrocephalus. Children with osteopertosis are at risk of having tetanic seizures due to hypocalcemia and secondary hyperparathyroidism. Continued bone formation and thickening interfere with medullary hematopoiesis which leads to bone marrow failure and compensatory extramedullary hematopoiesis occurs in various organs such as the spleen and liver, eventually resulting in hepatosplenomegaly, anemia, thrombocytopenia, granulocytopenia, and recurrent infections [1-3].

The diagnosis is established by: mutation analysis, clinical picture, and bone radiographs. The radiographs show a characteristic sign of increased bone density and bone on bone appearance, help in establishing the diagnosis. Patients with osteopetrosis require multidisciplinary team approach in their management in order to carefully evaluate and monitor the disease progression. The only treatment that has been proven to change the course of the disease is bone marrow transplantation of hematopoietic stem cells from human leukocyte antigen (HLA) identical donors [5]. However, bone marrow transplantation may not be beneficial for all patients because of the diversity of underlying causes of the disease [1]. Osteopetrosis prognosis is variable according to the type and severity of the disease. Reduced life expectancy is associated with the severe infantile type of osteopetrosis. As a result of bone marrow suppression, the majority of children die in the first decade of life [6]. Due to the rarity of this type of osteopetrosis, we would like to report this case.

## Case presentation

A 2-month-old female infant was admitted for respiratory infection, with a past medical history of respiratory distress in her first day of life that required hospitalization in the neonatal care. Sepsis workup was done and she received antibiotics. During the follow-up exam, thrombocytopenia and spasticity were observed. She was born after an uncomplicated pregnancy and was delivered at 38 gestational weeks. Birth weight was 2.5 kg. The parents are consanguineous as first degree cousins. They have no history of inherited genetic diseases, inherited hematological diseases, abortions or neonatal deaths.

General physical examination revealed spasticity of the upper and lower limbs with arching of the back and head lag (axial hypotonia). Her growth parameters were all less than the third percentile. She had a pale appearance. Abdominal exam showed a distended abdomen, hepatomegaly with the liver noted to be 2 cm below the costal margin, firm and smooth surface, splenomegaly with the spleen noted to be 5 cm below the costal margin. The funduscopic examination concluded visual impairment picture with no obvious cherry-red spots.

**Figure 1:**
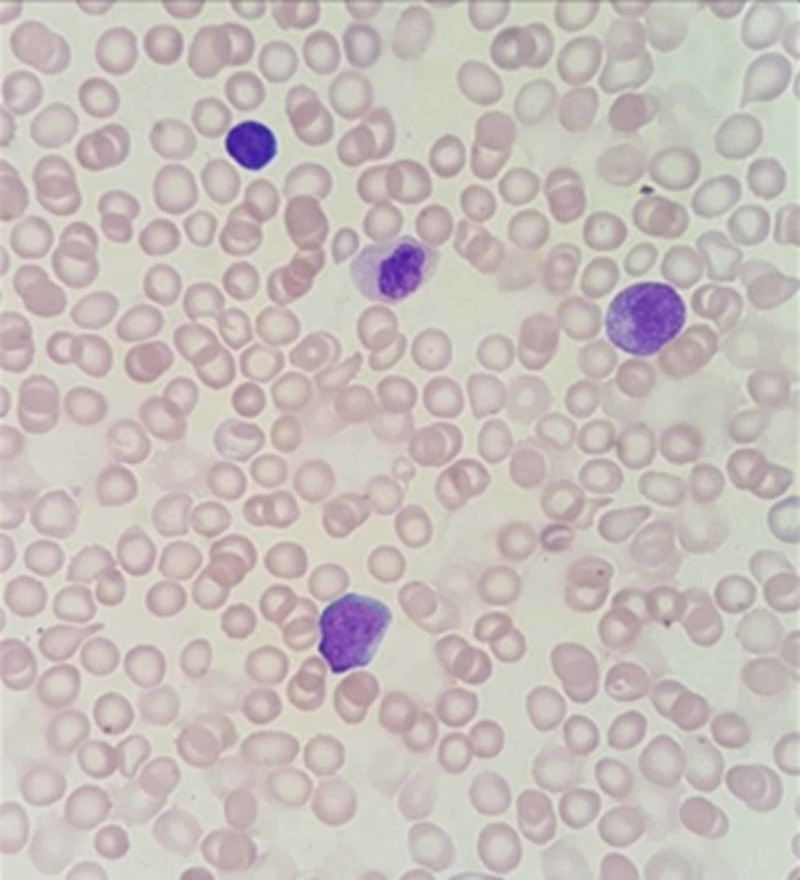
Increase in leukocytes with immature erythroids called normoblasts; some red blood cells appear as agglutinated cells with rouleaux formation

**Figure 2:**
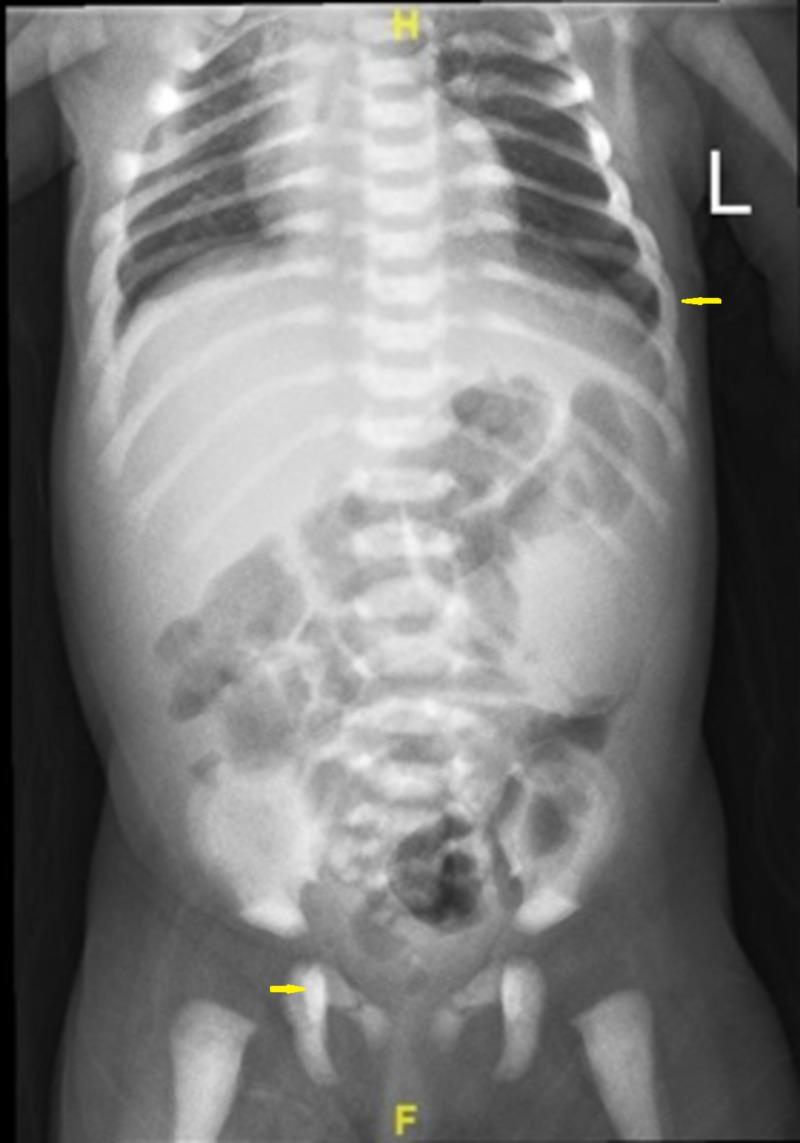
Generalized increase in bone density and widening of the ribs at the costochondral junction

**Figure 3:**
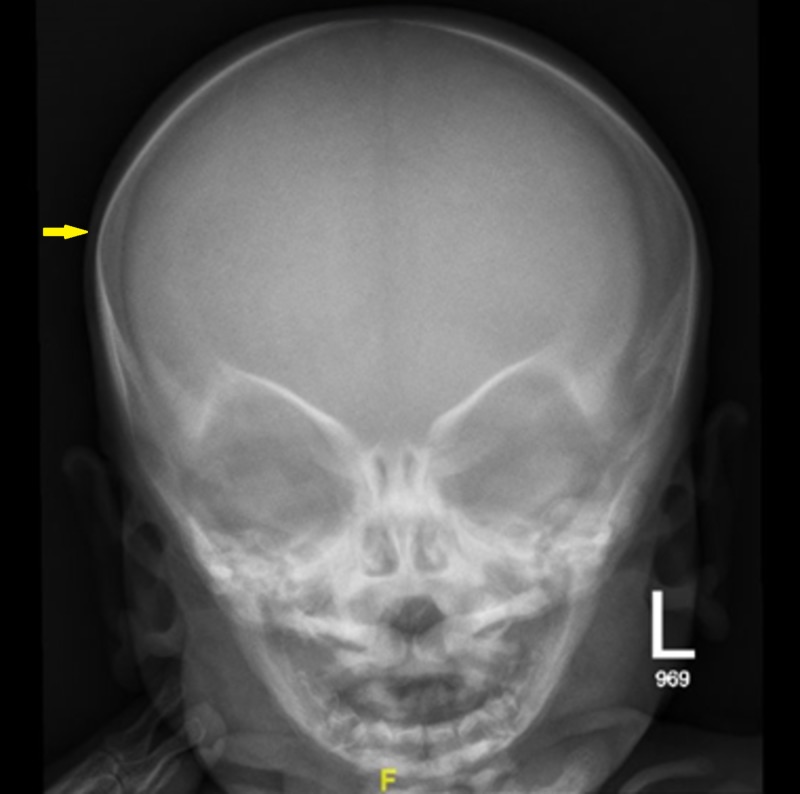
Skull radiograph showed normal scanned osseous structures regarding their density and cortical outline, with no evidence of any fracture line

**Figure 4:**
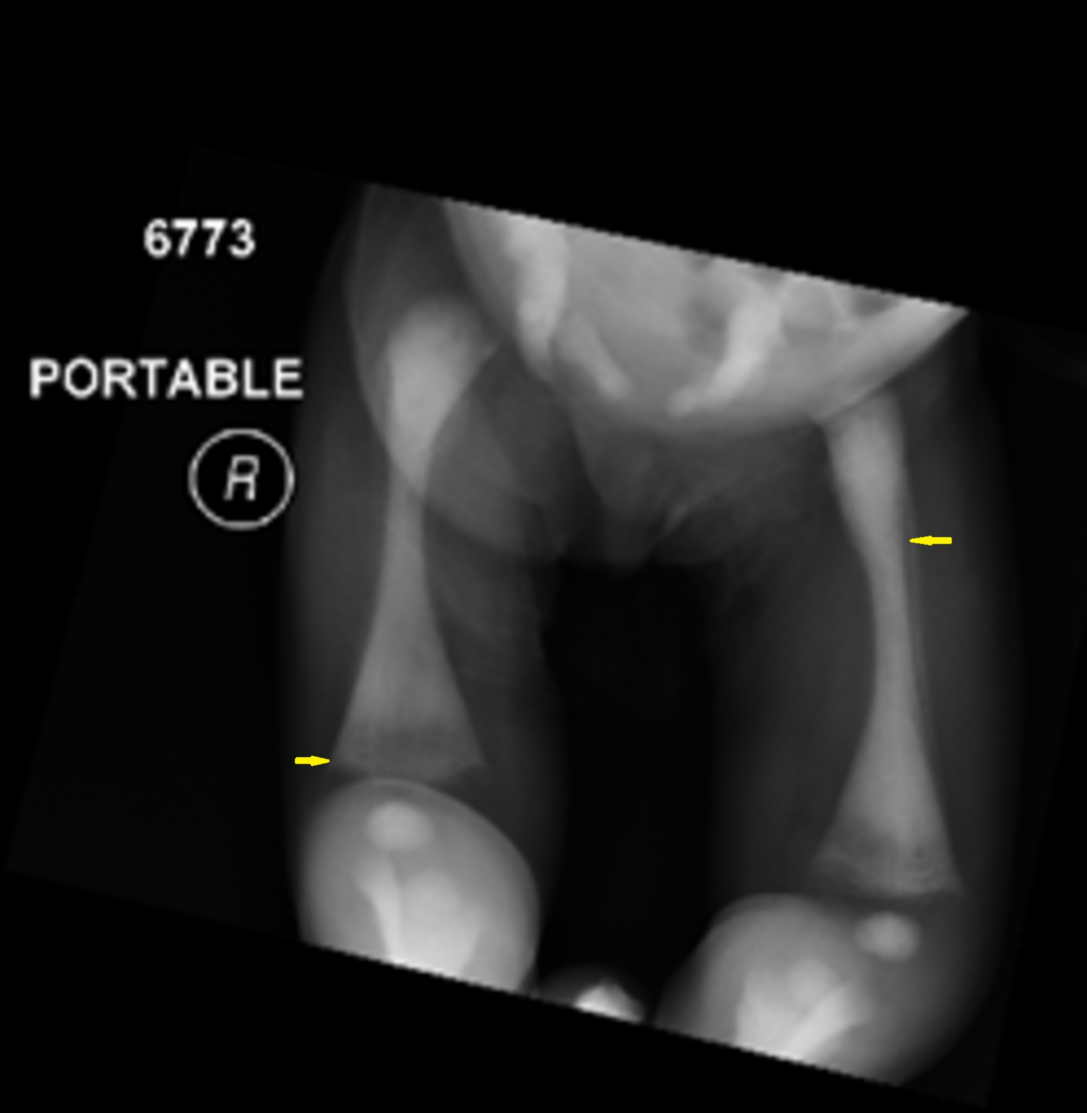
Increased sclerosis of both femurs with metaphyseal flaring

**Figure 5:**
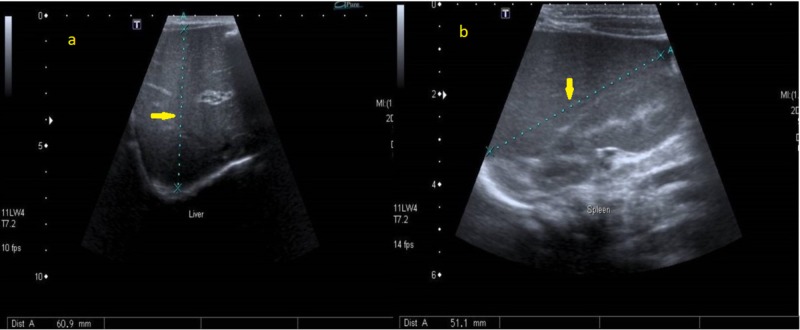
Abdominal ultrasound of the patient showed hepatosplenomegaly (a) Liver, (b) Spleen.

Laboratory examination yielded the following: hemoglobin was 8.7 g/dl, white blood cell count was 8.5 cells/mm3, platelet count was 54/mm3 and calcium level was 8.5 mg/dL. The biological analysis was mostly within normal limits. Peripheral blood films were significant for leukoerythrocytosis (Figure 1). Serology for toxoplasma, human herpesvirus 6, cytomegalovirus, and rubella was negative. Immunoglobulin (G, A, M) levels were normal. Lymphocyte subsets, urine and serum amino acid, and urine organic acid analyses were also normal. Skeletal radiographs revealed a generalized increase in bone density, increased sclerosis of both femurs with metaphyseal flaring, and widening of the ribs at the costochondral junction (Figures 2-4). Ultrasonographic examination of the abdomen confirmed the marked hepatosplenomegaly (Figure 5).

The primary diagnosis was an acquired congenital infection (TORCH) or a type of storage disease (like lipid storage or lysosomal diseases) but the diagnosis of osteopetrosis was confirmed by physical exam and laboratory findings. Genetic testing revealed a homozygous variant of OSTM1 which was identified in this patient. This is a known Saudi mutation (KSM) and should be considered pathogenic. Loss of function variants of OSTM1 are frequently the cause of autosomal recessive osteopetrosis (ARO). The patient was managed by full supportive measures with antibiotics, platelets, and PRBCS transfusion. Seizure was controlled with vigabatrin and Keppra, and the patient was referred to the pediatric hematology-oncology service for bone marrow transplantation as it is the only treatment for osteopetrosis. The patient spent four months in the neonatal intensive care unit and deceased due to cardio-pulmonary arrest secondary to pneumonia and severe respiratory complications.

## Discussion

In 1904, German radiologist Albers-Schönberg first described osteopetrosis. Clinical manifestations of osteopetrosis are extremely variable, ranging in severity from asymptomatic to a fatal course. It has a widely heterogeneous set of conditions that share the radiological feature of increased bone density due to differentiation or functional abnormalities in osteoclast [6].

Osteopetrosis has two major clinical types: the autosomal dominant adult (benign) type which is associated with milder symptoms that often occur in later childhood and adulthood, while patients with the autosomal recessive infantile (malignant) type present with severe symptoms in very early childhood and typically leads to a fatal course if untreated [1].

Our case is of the IMOP form. It is inherited as an autosomal recessive pattern that begins in utero and manifests at birth or within the first year of life. This form is more serious and carries high severity compared to the autosomal dominant [7-10]. It has an incidence of 1 in 250,000 births [11,4]. The main presenting features include; short stature, fractures, osteomyelitis, typical facial appearance due to macrocephaly, and frontal bossing [12]. Our patient presented at the age of two months with a history of recurrent fever, recurrent pneumonia, developmental delay, and infantile spasms, without bone fractures or osteomyelitis.

Medullary hematopoiesis is affected by the abnormal expansion of bone due to continued bone formation and thickening, resulting in bone marrow failure, life-threating pancytopenia, and compensatory extra-medullary hematopoiesis which lead to hepatosplenomegaly as found in our case.

The abnormally thickened bone can cause narrow cranial nerves foramina resulting in facial palsy, deafness, and blindness [13]. Primary seizures with normal calcium levels, hypotonia, developmental delay, retinal atrophy, and sensorineural deafness are signs of primary neurodegeneration that appear in some patients with ARO variants (neuropathic ARO) [13]. Approximately about 78% of individuals with ARO are affected by hearing loss [12]. Reported brain magnetic resonance imaging (MRI) findings include significantly delayed myelination, diffuse progressive cortical and subcortical atrophy, and bilateral atrial sub-ependymal heterotopias [14]. Brain MRI results of our patient suggested periventricular leukomalacia and foci of hemorrhage seen at the right parietal region. Our patient was found to have axial hypotonia, limb spasticity, infantile spasms, and visual impairment.

Diagnosing osteopetrosis is mostly relied on skeletal radiology which typically shows a marked increase in bone density with defective metaphyseal remodeling, and “a bone within a bone” appearance [11]. A “sandwich” appearance of the vertebrae due to alternating sclerotic and radiolucent transverse metaphyseal lines is also seen [6]. A femur X-ray was done for our patient and revealed an increased sclerosis of both femurs with metaphyseal flaring.

Differential diagnoses, storage diseases, and acquired congenital infection were ruled out due to laboratory test findings not supporting them. Bone marrow aspiration was done and malignancy ruled out. No gaucher cells or foamy histocytes were found.

Genetic testing can be used to confirm the diagnosis and distinguish between various osteopetrosis subtypes. It helps in obtaining information related to disease prognosis, response to therapy and recurrence risks [6]. Our patient genetic testing revealed a homozygous variant of OSTM1 gene, which is a known Saudi mutation of ARO.

Managing osteopetrosis patients’ required a multidisciplinary comprehensive approach to the various characteristic clinical issues such as neurological complications, bone defects, recurrent infections, hematological and metabolic abnormalities [15].

HSCT currently provides the main chance for IMOP to be cured; it should be done early before the irreversible neurological sequelae appear. 73% five-year disease-free survival is achieved by HSCT using HLA-identical donors [16].

Some patients with osteopetrosis variants that are not responsive to HSCT or before transplantation have been given interferon gamma 1b (IFNγ1b) therapy. It has been reported to improve the immune function, enhance bone resorption, and increase bone marrow space [17-18].

Providing genetic counseling is essential. Radiographs may be used for possible antenatal diagnosis in families with IMOP, therefore enabling HSCT before the age of three months to improve neurological outcomes [19]. However, the challenge and difficulty of radiographic assessment of the fetus in obtaining conclusive results, makes prenatal molecular diagnosis extremely desirable [20].

## Conclusions

Osteopetrosis prognosis is variable according to the type and severity of the disease. The severe infantile form of osteopetrosis is associated with reduced life expectancy, with most untreated children dying in the first decade as a complication of bone marrow suppression. IMOP should be considered as a rare differential of hepatosplenomegaly. Early diagnosis by clinical picture, imaging, and genetic testing is important to direct appropriate management in order to prevent disease progression before the irreversible neurological problems occur.

## References

[1] Lam DK, Sándor GKB, Holmes HI, Carmichael RP, Clokie CML (2007). Marble bone disease: a review of osteopetrosis and its oral health implications for dentists. J Can Dent Assoc.

[2] Goswami R (2016). Primer on the metabolic bone diseases and disorders of mineral metabolism. Indian J Med Res.

[3] Kalekar T, Sehrawat V (2017). Autosomal recessive infantile osteopetrosis: case report with radiological review. Int J Res Med Sci.

[4] Loria-Cortes R, Quesada-Calvo E, Cordero-Chaverri C (1977). Osteopetrosis in children: a report of 26 cases. J Pediatr.

[5] Wilson CJ, Vellodi A (2000). Autosomal recessive osteopetrosis: diagnosis, management, and outcome. Arch Dis Child.

[6] Stark Z, Savarirayan R (2009). Osteopetrosis. Orphanet J Rare Dis.

[7] Sunita M, Managutti A, Pragasm M (2012). Infantile osteomyelitis secondary to malignant osteopetrosis. J Maxillofac Oral Surg.

[8] Engiz O, Kara S, Bagrul D, Lahr G, Alioglu B, Arikan I, Bilge YD (2012). Infantile malignant osteopetrosis: a rare cause of neonatal hypocalcemia. J Pediatr Endocrinol Metab.

[9] Kurtoglu S, Hatipoglu N, Canpolat M (2009). Malignant infantile osteopetrosis presenting with neonatalypocalcemia. Erciyes Med J.

[10] Tolar J, Teitelbaum SL, Orchard PJ (2004). Osteopetrosis. N Engl J Med.

[11] Subramaniam A, Singh A, Chavan M, Kunte S (2008). Autosomal recessive osteopetrosis: case report of two siblings. Oral Radiol.

[12] Al-Tamimi YZ, Tyagi AK, Chumas PD, Crimmins DW (2008). Patients with autosomal-recessive osteopetrosis presenting with hydrocephalus and hindbrain posterior fossa crowding. J Neurosurg Pediatr.

[13] Dozier T, Duncan I, Klein A, Lambert P, Key L (2005). Otologic manifestations of malignant osteopetrosis. Otol Neurotol.

[14] Maranda B, Chabot G, Decarie JC, Pata M, Azeddine B, Moreau A, Vacher J (2008). Clinical and cellular manifestations of OSTM1-related infantile osteopetrosis. J Bone Miner Res.

[15] Ahmad I, Abbas SZ, Haque F, Rashid M, Ahmad SA (2006). Osteomyelitis of mandible - a rare presentation of osteopetrosis. Indian J Radiol Imaging.

[16] Driessen GJ, Gerritsen EJ, Fischer A (2003). Long-term outcome of haematopoietic stem cell transplantation in autosomal recessive osteopetrosis: an EBMT report. Bone Marrow Transplant.

[17] Key LL Jr, Ries WL, Rodriguiz RM, Hatcher HC (1992). Recombinant human interferon gamma therapy for osteopetrosis. J Pediatr.

[18] Lyseng-Williamson KA (2015). Interferon γ-1b in chronic granulomatous disease and severe malignant osteopetrosis: a guide to its use in the USA. Drugs Ther Perspect.

[19] Ogur G, Ogur E, Celasun B, Basȩr I, Ïmïrzalioǧlu N, Öztürk T, Alemdaroǧlu A (1995). Prenatal diagnosis of autosomal recessive osteopetrosis, infantile type, by X-ray evaluation. Prenat Diagn.

[20] Kulkarni ML, Marakkanavar SN, Sushanth S (2007). Osteopetrosis with Arnold chiari malformation type1 and brain stem compression. Indian J Pediatr.

